# Type B insulin resistance syndrome: a systematic review

**DOI:** 10.20945/2359-3997000000257

**Published:** 2020-05-27

**Authors:** Luizianne Mariano Martins, Virgínia Oliveira Fernandes, Manuela Montenegro Dias de Carvalho, Daniel Duarte Gadelha, Paulo Cruz de Queiroz, Renan Magalhães Montenegro

**Affiliations:** 1 Hospital Universitário Walter Cantídio Faculdade de Medicina Universidade Federal do Ceará Fortaleza CE Brasil Hospital Universitário Walter Cantídio, Faculdade de Medicina, Universidade Federal do Ceará (UFC), Fortaleza, CE, Brasil

**Keywords:** Anti-insulin receptor antibody, insulin resistance syndrome, diabetes, autoimmunity

## Abstract

A literature review on the clinical, laboratory, and treatment features of type B insulin resistance syndrome (TBIRS). Data from PubMed, the Virtual Health Library and Cochrane database were selected and analyzed using the REDCap application and R statistical program. From 182 papers, 65 were selected, which assessed 119 clinical cases, 76.5% in females and 42.9% in African-Americans, with an average age of 44 years. A common feature of TBIRS is co-occurrence of autoimmune diseases, such as systemic lupus erythematosus (most frequently reported). Hyperglycemia of difficult control was the mostly reported condition. Tests for anti-insulin receptor antibodies were positive in 44.2% of the cases. Disease management comprised fractional diet, insulin therapy (maximum dose given was 57 600 IU/day), plasmapheresis and immunosuppression with several classes of drugs, mainly glucocorticoids. Remission occurred in 69.7% of cases, in 30.3% of these spontaneously. The mortality rate was 15.38%. There was an inverse relationship between anti-insulin antibodies and remission (p = 0.033); and a positive correlation between combined immunosuppressive therapy and remission (p = 0.002). Relapse occurred in 7.6% of the cases. This rare syndrome has difficult-to-control diabetes, even with high doses of insulin, and it is usually associated with autoimmune diseases. Therapeutic advances using immunomodulatory therapy have led to significant improvements in the rate of remission.

## INTRODUCTION

Type B insulin resistance syndrome (TBIRS) is an especially rare autoimmune disorder with unknown prevalence, caused by immunoglobulin G polyclonal antibodies that antagonize the insulin receptor. This antagonism leads to an abnormal cellular and metabolic responses to insulin, marked by elevated levels of circulating insulin, while the type A insulin resistance syndrome is induced by genetic defects in the signaling system and the insulin receptor ( 1 - 3 ). This results in compensatory insulin hypersecretion, usually at levels at which normoglycemia cannot be maintained ( 1 ). Eventually, the receptor autoantibodies can present an insulin-like effect, leading to hypoglycemia ( 1 ).

It is generally described in middle-aged people and may be associated with another autoimmune condition. Diagnosis is given by positive anti-insulin receptor antibody finding. This syndrome is difficult to diagnose, as it may present as hyper- or hypoglycemia which is difficult to control. The triad of high fasting insulin levels associated with hyperadiponectinemia and normal or low triglycerides may be a clue to the diagnosis. Individuals who require insulin at doses greater than 3 U/kg/day and have concomitant autoimmune diseases should be investigated for TBIRS. It should also be noted that some cases can present with hypoglycemia or hyper-hypoglycemia raising the question of whether TBIRS should be investigated in diabetic patients with associated autoimmune disorders and poor glycemic control despite correct adherence to treatment.

Treatment includes glycemic control measures and sometimes immunosuppression, but there is no standard immunosuppression protocol, described in the literature, tested in randomized trials. Hospitalization is often required for administration of extremely high doses of insulin, or for monitoring severe and difficult to control hypoglycemia that is related to high mortality rates. Since this is a rare condition, there are few articles in the literature describing the clinical presentation, laboratory diagnosis and treatment options. This study is a systematic review of the literature on the main clinical, laboratory and therapeutic features of TBIRS.

## MATERIALS AND METHODS

### Literature search strategies

In May 2018 searches were performed in the PubMed, the Virtual Health Library and Cochrane electronic databases, without restrictions regarding date of publication. There are no Medical Subject Headings (MESH) terms to describe the syndrome, so the terms ‘“type B insulin resistance” and “type B syndrome” were used.

In addition, the bibliographic reference lists of all selected articles were manually reviewed to retrieve other potentially eligible publications.

### Inclusion and exclusion criteria

This systematic review adapted the recommendations of the MOOSE Guidelines (Meta-Analyses of Observational Studies). Hence, most of the studies selected were case reports. Full articles were included, regardless of the methodological approach used, in which patients obtained confirmation of the diagnosis of TBIRS or, even without treatment with anti-insulin receptor antibodies (AIRAs), had a clinical condition suggestive of TBIRS and responded to immunosuppressive therapy. These articles were published in English, Portuguese or Spanish. Articles that focused only on the molecular diagnosis and did not address the syndrome in humans were excluded. Duplicate articles or those for which the full text was not available were also excluded.

### Variables analyzed

Demographic and clinical features, laboratory findings, treatment and relapse data were analyzed. This included data on gender, age, body mass index (BMI), pre-existing conditions, antibody panel, anti-insulin response, acanthosis, episodes of hypoglycemia, glycated hemoglobin and triglyceride levels, ketosis, options for maximum dose of insulin, treatment options, remission and relapse. In the analysis of the therapeutic options, the treatment was divided into three phases and maintenance. Patients who did not respond to the medication in the first phase were moved to the second phase, and if they still did not respond, were moved to the third phase of treatment. In some cases, maintenance was carried out regardless of the number of phases. The criteria considered in most studies as indicating remission were reduction or suspension of the insulin dose, and reduction of AIRAs titers. Relapse cases were those in which the patients were in remission and needed to restart insulin use or to increase the daily dose, and in which patients presented with hypoglycemia without the use of hypoglycemic drugs.

### Statistical analysis

The data were initially organized in Microsoft Excel^®^ and later placed in the online Research Data Capture (REDCap^®^) application. The generated spreadsheets were analyzed using the same application and the R-3.5 statistical program (R Core Team, 2018, Vienna, Austria). In the descriptive statistics the mean, median, standard deviation and simple and relative frequencies were used, depending on the nature of the variable. Due to the limitations of the present review, the focus will be on descriptive statistics. Means, median (which is preferable), standard deviation, simple and relative frequencies were used, depending on the nature of the variable. When strictly necessary for multivariate analysis, binomial logistic regression was used. For simpler analysis, a combination of chi-square and Fisher’s exact test was used. Test results were considered significant when p < 0.05. No fixed/random effects were added as it is a collection of case studies. Among the characteristics of this type of study cluster was the absence of a control group, implying that relative risk calculations and odds ratios lost their meaning. Since most studies have n = 1, it was not possible to calculate the effect size either.

## RESULTS

**Figure 1:**
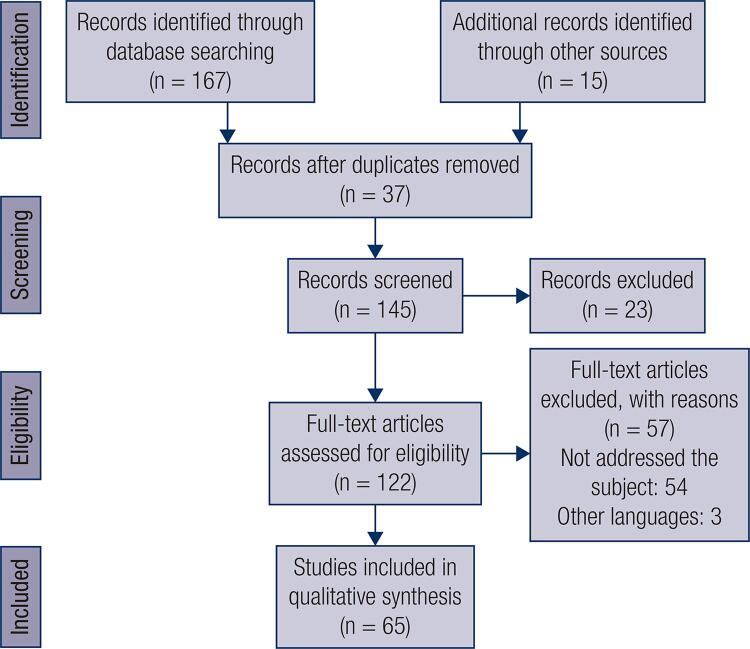
Study selection flowchart.

This review resulted in the selection of 65 articles covering 115 clinical cases of TBIRS, as shown in Figure 1 . The articles included were published by several countries: United States of America (29), Japan (14), China (5), France (4), United Kingdom (4), Korea (2), South Korea (1), Peru (1), Spain (1), India (1), Israel (1), Italy (1), Spain (1) and Romania (1).

### Demographic data

Females were predominantly affected (n = 91; 76.5%) by TBIRS. The ages of patients ranged from 8 months to 76 years, and the syndrome was more common during the fifth and sixth decades of life, with a mean age of 44 ± 16.9 years. Onset occurred at an earlier median age (42 years) in women and later in men (median age 59 years). The two earliest case were described in an 8-month-old child with unknown disease and a 1-year old child with T-cell immunodeficiency ( 2 ). It occurred most often in African Americans (n = 51; 42.9%), followed by Asians (n = 20; 16.8%), Caucasians (n = 11; 9.2%), Latins (n = 01; 0.8%) and Africans ( n = 1; 0.8%).

### Associated diseases

Among the associated diseases, the most common were autoimmune (n = 60; 52.2%), unknown (n = 43; 37.4%), infectious (n = 7; 6.1%) and lymphoproliferative (n = 2; 1.7%). Among the autoimmune diseases, the most frequent was systemic lupus erythematosus (SLE), which was present in up to 35,3% (n = 42) of the reported cases. No other associated disease was found in 37.4% (n = 43) of the cases. Some viral and bacterial diseases have also been associated with TBIRS, such as *Helicobacter pylori* ( 3 , 4 ), human immunodeficiency virus (HIV) ( 5 ), hepatitis C ( 6 , 7 ), and human T-cell lymphotropic virus ( 8 ). Two cases were associated with Hodgkin’s lymphoma ( 2 , 9 ). Type B insulin resistance syndrome was also associated with other autoimmune endocrinological disorders, such as Hashimoto’s thyroiditis ( 7 , 10 , 11 ) and type 1 diabetes mellitus ( 12 - 14 ).

### Laboratory analysis

The mean levels of glycated hemoglobin (HbA 1 c) were 10.8 ± 2.9%, and the highest was 18.7% ( Table 1 ). Lower levels of HbA1c were observed in cases that presented with hypoglycemia.

**Table 1:** Evaluation of laboratory parameters

	Evaluation of Laboratory Parameters			

	Investigated	Mean	Standard deviation	Range
Glycated hemoglobin	34.5%	10.8%	2.9%	5.1%-18.7%
Serum insulin (µU/mL)	19.3%	1309.1	2374.4	0.1-10 584.3
C-Peptide (ng/mL)	28.6%	13.9	31.3	0.1-63.0
Triglycerides (mg/dL)	16.0%	72.8	32.8	36,0-155.0
Leptin (pg/mL)	7.6%	7.9	11.6	0,1-35.6
IGF-1*	4.2%	-	-	-

* Insulin-like growth factor 1 (IGF-1) data were not included in the table as the analysis depends on the age group.

**Figure 2:**
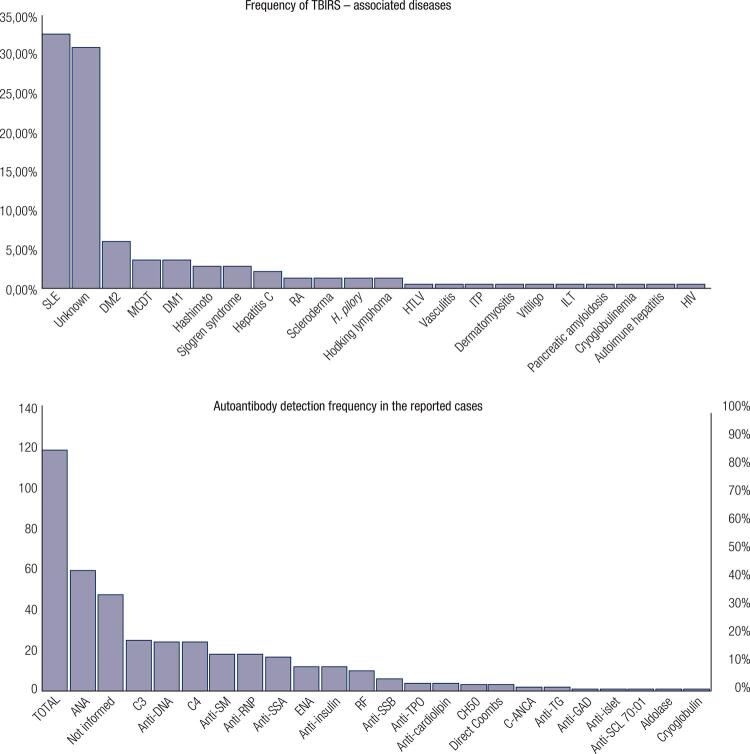
Frequency of associated diseases in tbirs and autoantibody detection frequency in the reported cases. TBIRS: type B insulin resistance syndrome; SLE: systemic lupus erythematosus; DM2: type 2 diabetes mellitus; MCTD: mixed connective tissue disease; DM1: type 1 diabetes mellitus; *H. pylori: Helicobacter pylori* ; RA: rheumatoid arthritis; HTLV: human T-cell lymphotropic virus infection; ITP: idiopathic thrombocytopenic purpura; ILT: T-cell immunodeficiency; HIV: human immunodeficiency virus infection; ANA: antinuclear antibody; C3: complement component 3; C4: complement component 4; ANTI-RNP: anti-ribonucleoprotein antibody; ANTI-SM: anti-Smith antibody; ENA: anti-extractable nuclear antigen antibodies; RF: rheumatoid factor; anti-SCL-70: topoisomerase I; Anti-TPO: anti-thyroid peroxidase antibody; CH50: total complement CH50; cANCA: cANCA: anti-neutrophil cytoplasmic antibody (classical cytoplasmic type); Anti-TG: anti-thyroglobulin; Anti-GAD: anti-glutamic acid decarboxylase autoantibodies.

Presence of AIRAs was described in 83.2% (n = 99) of the cases; it was not investigated in 17 patients (14.3%) due to technical difficulties in performing the test. Anti-insulin antibody was investigated in 43 cases (36.1%) and was positive in 44.2%. The most common panel of antibodies was found in SLE, with antinuclear antibody, anti-DNA and C3 and C4 complement consumption. The most commonly found autoimmunity test was that for antinuclear antibody (n = 60; 50.8%) ( Figure 2 ).

### Clinical features

Regarding BMI, 11.8% had low weight, 28.6% normal weight, and 7.6% were obese; 45.4% (n = 54) of the case reports did not report the BMI. *Acanthosis nigricans* was present in 64 patients (53.8%) and absent in 14.3%, but 31.9% (n = 38) of the cases reported did not include information on this clinical feature. Ketosis occurred in 11.8% of cases, but most reports (77.6%) did not indicate whether ketosis was present.

Isolated hyperglycemia was the most frequent clinical condition (n = 54; 45.4%), generally with postprandial glycemic rates higher than the fasting rates. Some patients could present with spontaneous remission of hyperglycemia and develop hypoglycemia ( 15 ). About 42.9% of the patients (n = 51) presented with hypoglycemia during the course of the disease, with isolated hypoglycemia in 21.8% of the cases. In these cases, there was no time pattern for its occurrence, and it can occur in both the fasting and postprandial periods. The other patients who developed hypoglycemia (21.1%) were on treatment or had previously received insulin treatment.

Amenorrhea and hyperandrogenism were present in a few cases ( 1 ). Some women presented with the severe clinical condition of associated hyperandrogenism, even with ovarian hyperthecosis or polycystic ovary syndrome. Type B insulin resistance syndrome was also found as a paraneoplastic manifestation of conditions such as Hodgkin’s disease and multiple myeloma ( 16 ). Some patients also had Raynaud’s syndrome ( 17 ).

### Treatment

#### Diet fractionation

Articles recommended diet fractionation in the treatment of both hyperglycemia and hypoglycemia, but no quantitative description was found ( 12 , 16 ).

#### Insulin therapy

The doses of insulin used in treatment ranged from 54 U/day to 57 600 U/day ( 39 ), with a median of 1747 U/day. The cases that demanded higher doses required hospitalization and the use of insulin therapy through an intravenous infusion pump.

#### Immunosuppression

**Figure 3:**
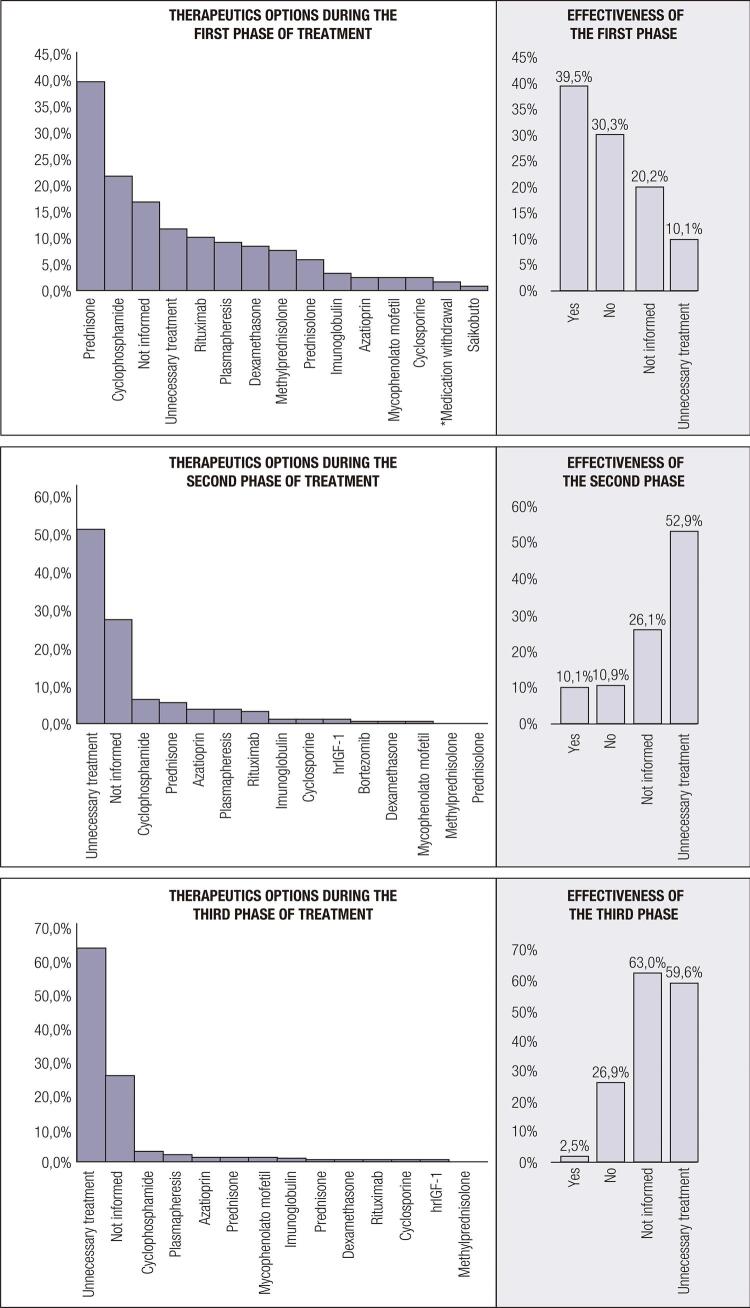
Therapeutic options and effectiveness during the first, second and third phases of treatment. * Withdrawal of the medication considered to be a trigger for development of the syndrome (case with withdrawal of ribavirin and interferon). hrIGF-1: human recombinant insulin-like growth factor I.

There was great variability regarding therapeutic options. The different treatment regimens are described in Figure 3 . In the first phase, the most frequently used medication was prednisone, followed by cyclophosphamide. In the second phase, the most frequently used medication was cyclophosphamide. In the maintenance phase, prednisone (n = 19; 16.0%) and azathioprine (n = 15; 12.6%) were the most frequently used, followed by mycophenolate mofetil (n = 2; 1.7%), leflunomide (n = 1; 0.8%), and cyclosporine (n = 1; 0.8%).

## Response to treatment

### Remission

Remission occurred in 83 cases (69.7%); of these, 20.5% presented spontaneous remission. Of the cases receiving treatment, during the first phase 2.0% (n = 2) achieved remission after withdrawal of the medication that had acted as a trigger of TBIRS. The time to reach remission ranged from 0.25 to 54 months, with a median of 4.0 months. There was a statistical association between the use of compound immunosuppressive treatments and remission (p = 0.002). There was a negative association between the presence of anti-insulin antibodies and remission (p = 0.033); however, to evaluate this specific association it was necessary to reduce the number of cases, as several articles in this review did not include information on anti-insulin antibody analysis.

### Relapse

Relapse occurred in 7.6% (n = 9) of the cases. The treatments used in those who experienced relapse were prednisone (2.5%, n = 3), azathioprine (1.7%, n = 2), cyclophosphamide (1.7%, n = 2), immunoglobulin (1.7%, n = 2), plasmapheresis (0.8%, n = 1). Some patients used more than one therapeutic option.

## Complications and mortality

### Microvascular complications

Nephropathy was present mainly in patients with SLE. However, few patients were submitted to a renal biopsy to determine if nephropathy was caused by decompensated diabetes or collagenosis. In patients with SLE and type B insulin resistance, proteinuria was common. Tsokos and cols. reported 7 cases of proteinuria out of 14 cases of type B insulin-resistant diabetes mellitus, and the complications of the lupus nephritis were diagnosed by renal biopsy in some of the cases ( 17 ). Diabetic retinopathy was rare ( 19 ). Cases of neuropathy have not been reported.

### Macrovascular complications

Two deaths as a result of atherosclerotic cardiovascular disease were reported, with 1 case due to acute myocardial infarction and 1 due to stroke.

### Mortality

In the series of cases in this review, 16 (15.38%) of the patients died, 4 (25%) of these due to intractable hypoglycemia. The others died from complications of SLE, acute myocardial infarction, stroke chronic kidney disease and breast cancer ( 2 ).

## DISCUSSION

Type B insulin resistance syndrome is a rare autoimmune condition caused by polyclonal antibodies that act against the insulin receptor, and can cause both insulin resistance and significant hyperglycemia, as well as hypoglycemia which is difficult to control ( 16 ), depending on receptor inhibition or activation ( 16 ). Diagnosis is based on presence of the AIRA, which is detected by immunoprecipitation, preferably during the hyperglycemic phase. In hypoglycemia, the antibody acts as a partial agonist and can be found in low titers ( 11 ). This is a technically difficult dosage to asses which led some authors to suspect the syndrome without considering the antibody.

Evidence of selective molecular interactions between insulin receptors and the major antigens of the histocompatibility system have been reported in human B lymphocytes ( 20 ). Insulin receptor autoantibodies have three mechanisms that lead to insulin resistance. Mechanism 1: Autoantibodies bind to insulin receptors and competitively inhibit insulin binding, preventing its action ( 11 ); Mechanism 2: They accelerate the receptor degradation rate ( 11 ); Mechanism 3: Paradoxically, these antibodies can act both as insulin agonists and antagonists, causing a biphasic response of hypo- and hyperglycemia in the same patient ( 11 ).

Studies with rodents showed that at high concentrations the antibodies act as a receptor antagonist, while at low titers they have a stimulatory effect, leading to a range of glycemic levels, from hypo- to hyperglycemia ( 16 ). Fasting rodents that received AIRAs showed hypoglycemia within 2 hours, and this persisted for up to 24 hours, suggesting an acute effect similar to that of insulin administration. In contrast, fed rodents that received AIRAs at high doses for many days became hyperglycemic, presumably due to desensitization of the receptor ( 16 ).

In general, the incidence of TBIRS is higher in middle-aged individuals, particularly those who were African American and female, with associated or hidden autoimmune diseases, who developed diabetes with a need for very high doses of insulin and glycemia which was difficult to control. During this review, it was noted that there is great variability in the descriptions of the physical examination, signs and symptoms, and the diagnostic investigation, making it difficult to standardize the evaluation of these patients. There are no pathognomonic signs that define the diagnosis ( 16 ). The most common clinical presentation described in the literature is the development of diabetes with persistent hyperglycemia despite the use of very high doses of insulin, demonstrating severe insulin resistance, associated with significant weight loss ( 16 ).

It should be noted that these individuals with TBIRS had a normal BMI or were thin and had low or normal levels of triglycerides, which was different from what should be expected in cases of metabolic syndrome. Despite significant insulin resistance, most patients in our collection of data had normal weight. The differential diagnosis considered all conditions that cause insulin resistance, including lipodystrophy. Another peculiar fact was that, although patients required very high doses of insulin for glycemic control, only 10.4% of the reported cases presented symptoms of ketosis.

The few measurements of C-peptide described in the literature showed that these patients could produce insulin and often did so at very high levels, but there was no glycemic control due to the action of the AIRA. More rarely, clinical presentation with life-threatening isolated hypoglycemia could also occur ( 21 ). The first case of hypoglycemia caused by AIRAs was described by Taylor and cols. in 1982 ( 22 ). In cases which presented with hypoglycemia there was no time pattern for when it occurs, which could be in both fasting and postprandial periods. In the differential diagnosis in those presenting with hypoglycemia, it was necessary to exclude insulinomas, adrenal insufficiency, hypothyroidism and IGF1, IGF2 producing tumors ( 23 ). Hypoglycemia and hyperglycemia were difficult to control even with treatment such as intravenous dextrose, glucagon, octreotide and corticosteroids. Some patients died due to the severity of the hypoglycemia and its lack of response to treatment. Some of these patients could present with spontaneous remission of hyperglycemia and then develop hypoglycemia ( 15 ), which made it difficult to manage clinically and led to death. Hypoglycemia that was difficult to treat led to four fatal outcomes ( 2 , 24 ).

Willard and cols. ( 16 ) suggest that the biochemical triad of markedly high concentrations of fasting insulin, hyperadiponectinemia and normal or low fasting triglyceride concentrations in an individual with acanthosis nigricans and an underlying autoimmune disease, can be considered a “functional” clinical definition of TBIRS. Low or normal triglyceride concentrations and hyperadiponectinemia can help distinguish this condition from those more commonly associated with insulin resistance ( 9 ).

In this review, acanthosis nigricans was present in most cases (54.8%). The association of acanthosis nigricans with multiple disorders characterized by insulin resistance suggests that hyperinsulinemia plays a key role in its development. Elevated insulin levels can stimulate proliferation of keratinocytes and dermal fibroblasts through the interaction with the IGF-1 receptor, resulting in the plaque-like lesions that characterize the disorder ( 25 ). It is speculated that other antibodies can be related to the genesis of acanthosis nigricans when there is no hyperglycemia; however, the mechanisms that determine the presence or absence of acanthosis are still unclear.

Patients with AIRAs usually have a coexisting autoimmune disorder (50%), most commonly SLE or undifferentiated autoimmune syndromes ( 26 ). Huang and cols. described how the presence of AIRAs can be an indication of active SLE ( 19 ); however, Rosenstein and cols. investigated the occurrence of AIRAs in patients with SLE or early undifferentiated connective tissue disease who did not present dysglycemia, and found no association ( 26 ). Therefore, patients with immunological diseases who did not present symptoms of dysglycemia should not be routinely screened for the presence of AIRAs. Patients with SLE and AIRAs had a poor prognosis, and most died from SLE.

Treatment was generally based on insulin therapy, diet fractionation and immunosuppressive therapy. The doses of intravenous insulin given were very high, and in one case reached 57 600 U/day ( 18 ); such amounts of insulin can only be infused intravenously using a continuous infusion pump, and this impairs the discharge of these patients.

Regarding immunosuppressive treatments, several strategies were adopted. Some patients showed remission with corticosteroid monotherapy ( 19 ). High-dose corticosteroids apparently have a more immediate efficacy, but no study suggests that long-term therapy has any benefit ( 11 ). Plasma glycemia can transiently worsen at the start of the treatment ( 19 ).

Other treatment strategies focused on control of the underlying systemic disease and, depending on their severity, included immunosuppressive therapies. Plasmapheresis, corticosteroids or cytotoxic agents have only short-term benefits. Willard and cols. state that mycophenolate mofetil, cyclophosphamide, azathioprine and various regimens of glucocorticoids as monotherapy or in combination also did not show a consistent clear benefit ( 9 ). There are few data about the use of intravenous immunoglobulin for treatment, but this therapy has been used in some cases.

In 2010, after studying a series of 14 patients which they followed up and a cohort of 24 other cases, the National Institutes of Health (NIH) developed a treatment protocol containing rituximab, cyclophosphamide, and steroids ( 27 ) that led to a significant reduction in the previously reported mortality rates ( 9 ). This was the largest longitudinal cohort of affected patients yet reported. Based on the compelling data from the NIH, combination therapy including cyclophosphamide is recommended to reduce the risk of hypoglycemia and, consequently, the mortality rate ( 9 ). An important finding regarding drug selection in the NIH protocol is that most patients in the cohort had lupus nephritis, an indication for treatment with cyclophosphamide.

Our analysis shows that adoption of a treatment protocol that includes corticosteroids and immunosuppressants (azathioprine, cyclophosphamide, cyclosporine, mycophenolate mofetil) was positively correlated with remission. Currently, it is not known whether maintenance therapy with azathioprine is essential to prevent relapse, or for how long maintenance therapy should be continued ( 9 ).

Hypoglycemia is indicative of a worse prognosis for the disease, and was found to be the main cause of death in our review. The overall mortality rates for this review differed from those found in the NIH case series (15.8% *vs.* 50%, respectively). This difference is probably due to the fact that the NIH received a limited number of patients, who were usually more severely affected. In addition, the NIH review was carried out with a cohort study while our review was a cross-sectional study from cases reported in the literature.

This is the review study that included the largest number of reported cases in the literature so far, and cataloged and analyzed more variables simultaneously. In our analysis, the presence of anti-insulin antibodies correlated negatively with remission of TBIRS. However, this analysis was hampered by the fact that several case reports did not include information on this antibody, and therefore the sample size had to be reduced to include just those cases where such information was available. The limitations of this study should be considered, as it was based on the analysis of case reports and series, and the data are not available in a homogeneous and clear manner and different variables were analyzed. The cases were analyzed quite differently regarding laboratory tests, without standardization and sometimes several parameters not being evaluated, as shown in Table 1 . Also, an important limitation is that many studies do not mention the treatment used, as it can be seen in Figure 3 . These studies were not excluded because they allowed to evaluate the clinical characteristics of a rare syndrome. The studies are listed in Table 2 . There are no financial conflicts of interest to disclose.

**Table 2:** Studies included in this systematic review

Ref.	Title	Year	Locality	Report	N
4	Saibokuto as a Possible Therapy for Type B Insulin Resistance Syndrome: The Disappearance of Anti-insulin Receptor Antibody and a Marked Amelioration of Glycemic Control by Saibokuto Treatment	2018	Japan	CR	1
29	Antibody-Mediated Extreme Insulin Resistance: A Report of Three Cases	2018	USA	CR	3
11	Immunosuppressive Therapy in Treatment of Refractory Hypoglycemia in Type B Insulin Resistance: A Case Report	2017	USA	CR	1
12	Intractable Hypoglycemia in the Setting of Autoimmune Overlap Syndrome	2017	UK	CR	1
28	Rituximab for the treatment of type B insulin resistance syndrome: a case report and review of the literature	2017	Japan	CR + R	1
30	Successful treatment of type B insulin resistance with mixed connective tissue disease by pulse glucocorticoids and cyclophosphamide	2017	China	CR	1
49	Effect of Liraglutide on Type B Insulin Resistance Syndrome and Insulin Allergy in Type 2 Diabetes: A Case Report.	2017	Japan	CR	1
3	Type B insulin resistance syndrome with Scleroderma successfully treated with multiple immune suppressants after eradication of Helicobacter pylori infection: a case report	2016	China	CR	1
8	Type-B Insulin Resistance in Peru	2016	Peru	CR	2
9	Diabetic Ketoacidosis Without Diabetes.	2016	USA	CR	1
16	Type B insulin resistance syndrome	2016	USA	R	0
19	A systemic lupus erythematosus patient presenting as type B insulin resistance complicated with cryoglobulinemia	2016	China	CR	1
65	Successful treatment of type B insulin resistance with rituximab	2015	UK	CR	1
42	Efficacy of oral glucocorticoid and cyclosporine in a case of rituximab-refractory type B insulin resistance syndrome	2015	Japan	CR	1
23	Type B Insulin-resistance syndrome: a cause of reversible autoimmune hypoglycemia	2014	France	CR	1
7	Type 1 diabetes mellitus with dual autoimmune mechanism related to pegylated interferon and ribavirin treatment for chronic HCV hepatitis	2013	Romania	CR	1
21	Type B insulin resistance syndrome induced by systemic lupus erythematosus and successfully treated with intravenous immunoglobulin: case report and systematic review	2013	China	CR+SR	1 (CR) + 67 (SR)
38	Type B insulin-resistance syndrome presenting as autoimmune hypoglycemia, associated with systemic lupus erythematosus and interstitial lung disease.	2013	Korea	CR	1
48	Successful control of a case of severe insulin allergy with liraglutide	2013	Japan	CR	1
13	Association of type B insulin resistance and type 1 diabetes resulting in ketoacidosis	2012	France	CR	1
14	Type B insulin resistance syndrome with diabetic ketoacidosis	2012	SK	CR	1
5	Type B insulin resistance syndrome associated with an immune reconstitution inflammatory syndrome in an HIV-infected woman	2011	France	CR	1
43	Autoimmune hypoglycemia in a patient with characterization of insulin receptor autoantibodies	2011	Korea	CR	1
55	Recurrent hypoglycemia during pregnancies in a woman with multiple autoantibodies including anti-insulin receptor antibody and anti-platelet antibody, whose serum lowered murine blood glucose levels and phosphorylated insulin receptor of CHO-IR cells	2011	Japan	CR	1
60	A woman with severe lupus nephritis and difficult to control diabetes mellitus	2011	USA	CR	1
27	Treatment of type B insulin resistance: a novel approach to reduce insulin receptor autoantibodies	2010	USA	C	38
32	Type B insulin resistance complicated with systemic lupus erythematosus	2010	Japan	CR	1
33	Type b insulin resistance syndrome	2010	Spain	CR	1
56	Systemic lupus erythematosus presenting as hypoglycaemia with insulin receptor antibodies and insulin autoantibodies.	2009	China	CR	1
6	Type B insulin resistance developing during interferon-alpha therapy	2009	USA	CR	1
31	Type B insulin resistance in a systemic lupus erythematosus patient	2009	India	CR	1
59	Type B insulin resistance syndrome induced by increased activity of systemic lupus erythematosus in a hemodialysis patient	2008	Japan	CR	1
10	Regression of acanthosis nigricans correlates with disappearance of anti-insulin receptor autoantibodies and achievement of euglycemia in type B insulin resistance syndrome	2007	USA	CR	1
34	A patient with type B insulin resistance syndrome, responsive to immune therapy	2007	USA	CR	1
35	Paradoxical elevation of high-molecular weight adiponectin in acquired extreme insulin resistance due to insulin receptor antibodies	2007	UK	OST	7
44	Type B insulin resistance syndrome associated with systemic lupus erythematosus	2007	USA	CR	1
50	Severe hypoglycaemia in a person with insulin autoimmune syndrome accompanied by insulin receptor anomaly type B	2007	Japan	CR	1
36	Successful treatment of Type B insulin resistance in a patient with otherwise quiescent systemic lupus erythematosus	2005	UK	CR	1
1	Treatment of systemic lupus erythematosus-associated type B insulin resistance syndrome with cyclophosphamide and mycophenolate mofetil	2003	USA	CR	1
2	Clinical course of the syndrome of autoantibodies to the insulin receptor (type B insulin resistance): a 28-year perspective	2002	USA	RC	24
26	The prevalence of insulin receptor antibodies in patients with systemic lupus erythematosus and related conditions	2001	USA	C	38
45	Anti-insulin receptor autoantibodies in a patient with type B insulin resistance and fasting hypoglycemia	2000	Japan	CR	1
51	A case of chronic hepatitis C developing insulin-dependent diabetes mellitus associated with various autoantibodies during interferon therapy	2000	Japan	CR	1
18	Clinical challenges of type B insulin resistance: a case study	1998	USA	CR	1
39	Systemic lupus erythematosus with acanthosis nigricans, hyperpigmentation, and insulin receptor antibody	1997	USA	CR	1
25	Absence of acanthosis nigricans in a patient with the type B syndrome of insulin resistance and preexisting diabetes	1996	USA	CR	1
64	Combination of glomerulonephritis with diabetic glomerulopathy in a patient with diabetes mellitus due to autoantibody to insulin receptor	1994	Japan	CR	1
15	Insulin resistance and hypoglycemia in a patient with systemic lupus erythematosus: description of antiinsulin receptor antibodies that enhance insulin binding and inhibit insulin action	1991	Italy	CR	1
37	Insulin resistance - mechanisms, syndromes, and implications	1991	USA	R	0
40	Insulin resistance and hyperinsulinemia induce hyperandrogenism in a young type B insulin-resistant female	1991	USA	CR	1
20	Hypoglycaemia induced by antibodies to insulin receptor following a bone marrow transplantation in an immunodeficient child	1989	France	CR	1
22	Systemic lupus erythematosus presenting as hypoglycaemia with insulin receptor antibodies	1989	USA	CR	1
41	Atypical antiinsulin receptor antibodies in a patient with type B insulin resistance and scleroderma	1989	USA	CR	1
54	Hyperinsulinemia due to impaired insulin clearance associated with fasting hypoglycemia and postprandial hyperglycemia: an analysis of a patient with antiinsulin receptor antibodies	1989	Japan	CR	1
57	Autoantibodies to the insulin receptor as a cause of autoimmune hypoglycemia in systemic lupus erythematosus	1988	USA	CR	1
46	Successful immunosuppressive therapy in a patient with autoantibodies to insulin receptors and immune complex glomerulonephritis	1987	USA	CR	1
53	Antibodies to insulin receptor followed by anti-idiotype: antibodies to insulin in child with hypoglycemia	1987	Israel	CR	1
52	Antiinsulin Receptor Antibodies in an Insulin-Dependent Diabetic May Arise as Autoantiidiotypes	1986	USA	CR	1
17	Lupus Nephritis and Other Autoimmune Features in Patients with Diabetes Mellitus Due to Autoantibody to Insulin Receptors	1985	USA	C	14
58	A connective tissue disease complicated by insulin resistance due to receptor antibodies: report of a case with high titer nuclear ribonucleoprotein antibodies	1982	USA	CR	1
47	Hypoglycemia associated with antibodies to the insulin receptor	1982	USA	CR	1
62	Insulin resistance due to receptor antibodies: a complication of progressive systemic sclerosis.	1980	USA	CR	1
63	Treatment by plasma exchange of a patient with autoantibodies to the insulin receptor	1979	USA	CR	1
24	The evolving clinical course of patients with insulin receptor autoantibodies: spontaneous remission or receptor proliferation with hypoglycemia	1978	USA	C	3
61	Successful immunosuppressive therapy in insulin resistant diabetes caused by anti-insulin receptor autoantibodies	1977	Japan	CR	1

Ref: references; CR: case report; RC: retrospective cohort; R: review; SR: systematic review; OST: observational study transversal; C: cohort; USA: United States of America; SK: South Korea; UK: United Kingdom.

In conclusion, type B insulin resistance syndrome is a rare immunological condition that can be associated with other underlying diseases. Therefore, identification of TBIRS presupposes that there is an active search for hidden underlying diseases. Based on the information available in the literature, a broad panel of antibodies should be used for rheumatologic and autoimmune diseases, and the search for anti-insulin antibodies should always be included, as these correlated with remission in this series of cases.

Remission can occur spontaneously or be secondary to a specific treatment. During treatment, the hyperglycemia can progress to hypoglycemia which is difficult to control, and the use of continuous glucose-monitoring systems is important. Combined treatment with corticosteroids and immunosuppressants was shown to have a more positive outcome. Standardization of parameters assessed in new reports could lead to more reliable studies in the future.
